# Declining health risk exposure among Chicago public high school students: Trends from the Youth Risk Behavior Survey 1997–2017

**DOI:** 10.1016/j.pmedr.2020.101161

**Published:** 2020-07-10

**Authors:** Jacqueline Korpics, Audrey Stillerman, Keiki Hinami, Sadhana Dharmapuri, Joseph Feinglass

**Affiliations:** a Cook County Health and Northwestern Feinberg School of Medicine, 1950 W Polk St, Chicago, IL 60612, United States; b Office of Community Engagement and Neighborhood Health Partnerships, University of Illinois at Chicago, 818 S. Wolcott Ave, Chicago, IL 60612, United States; c Collaborative Research Unit, Cook County Health, 1950 W Polk St, Chicago, IL 60612, United States; d Division of Adolescent Medicine, Cook County Health, 1950 W Polk St, Chicago, IL 60612, United States; e Division of General Internal Medicine, Northwestern University, 750 N Lakeshore Dr. 10th Floor, Chicago IL 60611, United States

**Keywords:** YRBS, Youth Risk Behavior Survey, CDC, Centers for Disease Control and Prevention, CPS, Chicago Public High School, MTF, Monitoring the Future National Survey, ACEs, Adverse Childhood Experiences, Teenage, Risk behaviors, Health behaviors, Multiple risk, Substance use, Mental health, Suicide risk, Violence, Victimization, Sexual health, Childhood adversity, Adolescent

## Abstract

There have been improvements nationally in teenagers' self-reported health risk since the 1990s. This study provides an overview of trends in substance use, sexual health, violence and victimization, and suicide risk among Chicago Public High School (CPS) students over a 20-year period. We compared responses to 29 identically worded items from the 1997, 2007, and 2017 Chicago Youth Risk Behavior Survey (YRBS) in the four domains. We show changes in responses across individual items, mean changes across the four domains, and change in the proportion of students with highest risk exposure (≥10 affirmative responses). Analyses control for CPS students’ grade, sex, and race/ethnicity. Reductions in substance use, sexual health risk, and violence and victimization (30, 40% and 40% in the mean number of affirmative responses, respectively) were observed. Suicide risk showed an initial improvement from 1997 to 2007, only to worsen by 2017 and show little difference from 1997. There was an approximate 70% decrease in the likelihood of being in the high multiple risk category (≥10 affirmative responses) in 2017 compared to 1997 (OR 0.33; CI 0.22-0.49). In alignment with national trends, our study documents significant improvement in Chicago public high school students’ long-term health risk exposure over the 20-year study period, with the notable exception of suicide risk. This study emphasizes the need to invest in strategies both inside and outside of the classroom to mitigate the effect of adversity and promote protective factors, which are at the root of academic success and overall wellbeing.

## Highlights

• There has been national improvement in teen self-reported health risk since the 1990s.
• Chicago is aligned with the nation in documenting improvements in teen health risk.
• Changes in teen risk align with academic achievement and changing demographics.
• Suicide risk is a notable exception to improvements in teen health risk.
• Mitigating the effects of adversity and promoting resilience must be a priority.

## 1 Introduction

Recently, several worrying trends involving teenagers have attracted national attention to Chicago. These include spikes in violence involving firearms (Fitzpatrick et al., 2019), widespread use of fentanyl-adulterated heroin and other illegal substances (The Drug Enforcement Administration (DEA), 2019), and youth homelessness (Putnam-Hornstein et al., 2017). In addition, Chicago saw a reduction in mental health services due to city mental health center closures as well as state budget shortfalls in 2016, closure of 50 schools almost exclusively in poorer communities of color in 2013, and the migration of middle-class minority families away from the city to the suburbs, particularly between 2000 and 2010 (Acs et al., 2017, Pendall, 2018). The impact of these developments on the health and well-being of Chicago high school students is still unfolding.

Health risk behaviors and exposures often emerge during childhood and adolescence, may extend into adulthood, and have impacts across the lifespan (Kann et al., 2017, Miech et al., 2019). Risk behaviors and exposures often occur together, and there is evidence that multiple risk behaviors are associated with effects beyond the additive effects of an individual risk behavior (Borodovsky and Krueger, 2019, de la Haye et al., 2014, Hale and Viner, 2016, Hale and Viner, 2012, Spring et al., 2012, Zwieg et al., 2002). Teenagers who experience multiple health risk exposures may be very different in terms of common vulnerability or protective factors from those teens who report only one or few risks (Borodovsky and Krueger, 2019, de la Haye et al., 2014, Hale and Viner, 2016, Hale and Viner, 2012, Spring et al., 2012, Zwieg et al., 2002). Vulnerability factors for teens include unmet social needs and adverse childhood experiences (ACEs), such as abuse, neglect, racism and discrimination, and community violence, which, for children living in Chicago’s South and West Side, includes the unfortunately common experience of witnessing gun violence (Felitti et al., 1998, Stillerman, 2018, Sonu et al., 2019). Insufficiently buffered by protective factors such as strong social support, experiences of adversity before age 18 are associated with poor physical and mental health, and even premature mortality, due to their deleterious impact on the stress response system, brain development, and overall physiology (Felitti et al., 1998, Stillerman, 2018, Sonu et al., 2019).

This study was undertaken as part of epidemiological monitoring of teenage health in Chicago. Our aim was to provide a historical context for recent trends in selected teenage health risks. The study examines changes in responses to the Centers for Disease Control and Prevention’s (CDC) Youth Risk Behavior Survey (YRBS) among Chicago Public High School (CPS) students over a 20-year period, using data from 1997, 2007, and 2017. We created an index composed of 29 identically worded items used in each survey wave selected to represent four domains: substance use, sexual health, violence and victimization, and suicide risk. We choose these domains because they are the most salient public health issues for our Chicago high school students and are closely linked to adverse childhood experiences (Felitti et al., 1998, Stillerman, 2018, Sonu et al., 2019). We present changes in these 29 items and four domains across the three YRBS study waves. Using 1997 as a benchmark, we also analyze the extent to which changes across all domains reduced the proportion of multiple health risk respondents in Chicago.

## 2 Methods

### 2.1 The Youth Risk Behavior Survey (YRBS)

This study used the CDC’s YRBS, which is administered to high school students across the United States, including CPS students, every two years during odd-numbered years (Kann et al., 2017). The YRBS is administered by school districts in order to assess the health risk behaviors and exposures most significantly associated with morbidity and mortality in teenagers. The YRBS sampling methodology has been described by the CDC elsewhere (Brener et al., 2013). The survey questions have demonstrated good test–retest reliability (Brener et al., 2013). A scientifically drawn sample, proper documentation of the sampling process, and an overall response rate greater than or equal to 60% were required to be publicly reported as weighted YRBS data (Brener et al., 2013). The Chicago YRBS samples used in this study are surveys of public high school students in Grades 9 to 12 in 1997, 2007, and 2017. YRBS data are presented as weighted estimates of Chicago’s public high school population in that year (Fitzpatrick et al., 2019, Kann et al.,1997, Eaton et al., 2007). Use of these data, which are de-identified and publicly available, are exempt from IRB review.

### 2.2 Identification of study risk factor items

We selected 29 identically worded YRBS items used across all three Chicago CPS survey waves, with items sorted by four domains of substance use, sexual health risk, violence and victimization, and suicide risk (shown in Table 2). The items and domains selected were a priori considered to best reflect a valid and broad picture of changes in student experiences over the 20-year interval. We selected six questions for violence/victimization, 14 questions about substance use, five questions about sexual health, and four questions about suicide risk. Each of these 29 YRBS questions was available in dichotomized form from the CDC, coded to indicate affirmative responses. Scales were computed as the sum of affirmative responses. Cronbach alpha across the 29 items was 0.88 in the baseline year 1997, and 0.87 across all three years. Cronbach alpha for the four subscale scores ranged from 0.70 to 0.81.

**Table 1:** Chicago Public High School Weighted Population Percentages and Response Rates in the 1997, 2007, and 2017 Youth Risk Behavior Survey (YRBS).

	1997 YRBS	2007 YRBS	2017 YRBS
Total Unweighted Respondents	1,423	1,118	1,883
Total Weighted CPS High School Population	97,458	88,033	77,313
Non-Hispanic White	8.1	10.2	11.4
Non-Hispanic Black	47.8	49.6	33.8
Hispanic	34.7	35.3	48.0
Other Race and Ethnicity	9.4	4.9	6.8
Female	54.3	52	51.4
Grade 9	34.1	32.6	25.9
Grade 10	28.5	26.2	25.4
Grade 11	21.0	21.3	24.7
Grade 12	16.0	19.5	23.5
School YRBS Response Rate (%)	95	96	97
Student YRBS Response Rate (%)	72	72	75
Overall YRBS Response Rate (%)	68	70	73

**Table 2:** Weighted Response Rates for 29 Youth Risk Behavior Survey Questions by Chicago Public High School Students in 1997, 2007, and 2017.

	**Percent Affirmative Respondents**		
	**1997**	**2007**	**2017**
**Violence and Victimization**			
**Q12 During the past 30 days, on how many days did you carry a weapon such as a gun, knife, or club? (One or more days)	23.6	17.8	11.9
**Q13 During the past 30 days, on how many days did you carry a weapon such as a gun, knife, or club on school property? (One or more days)	12.4	5.8	3.4
Q15 During the past 30 days, on how many days did you not go to school because you felt you would be unsafe at school or on your way to or from school? (One or more days)	13.9	12.3	10.0
*Q16 During the past 12 months, how many times has someone threatened or injured you with a weapon such as a gun, knife, or club on school property? (One or more times)	12.9	12.8	7.7
**Q17 During the past 12 months, how many times were you in a physical fight? (One or more times)	40.7	39.8	24.7
*Q18 During the past 12 months, how many times were you in a physical fight on school property? (One or more times)	17.9	17.4	10.0
**Substance Use**			
**Q30 Have you ever tried cigarette smoking, even one or two puffs? (Yes)	70.5	57.6	27.3
**Q32 During the past 30 days, on how many days did you smoke cigarettes? (One or more days)	26.8	13.2	6.0
Q33 During the past 30 days, on the days you smoked, how many cigarettes did you smoke per day? (Smoked more than 10 cigarettes per day)	4.8	7.7	5.9
*Q40 During your life, on how many days have you had at least one drink of alcohol? (One or more days)	71.2	71.4	57.3
**Q41 How old were you when you had your first drink of alcohol other than a few sips? (Before age 13)	35.0	25.1	16.8
**Q42 During the past 30 days, on how many days did you have at least one drink of alcohol? (One or more days)	36.9	38.9	23.9
Q46 During your life, how many times have you used marijuana? (One or more times)	44.8	44.0	43.8
Q47 How old were you when you tried marijuana for the first time? (Before age 13)	12.5	13.0	8.1
Q48 During the past 30 days, how many times did you use marijuana? (One or more times)	23.6	21.7	24.7
Q49 During your life, how many times have you used any form of cocaine, including powder, crack, or freebase? (One or more times)	5.1	5.9	6.6
Q50 During your life, how many times have you sniffed glue, breathed the contents of aerosol spray cans, or inhaled any paints or sprays to get high? (One or more times)	13.0	9.6	9.7
Q55 During your life, how many times have you taken steroid pills or shots without a doctor’s prescription? (One or more times)	4.2	4.0	5.7
Q57 During your life, how many times have you used a needle to inject any illegal drug into your body? (One or more times)	2.4	2.4	4.1
Q58 During the past 12 months, has anyone offered, sold, or given you an illegal drug on school property? (Yes)	28.4	32.9	32.3
**Sexual Health Risk**			
**Q59 Have you ever had sex? (Yes)	53.9	56.9	39.0
*Q60 How old were you when you had sexual intercourse for the first time? (Before age 13)	12.8	11.5	5.5
**Q61 During your life, with how many people have you had sexual intercourse? (4 or more persons)	19.9	18.1	9.8
*Q62 During the past 3 months, with how many people did you have sexual intercourse? (One or more persons)	37.7	39.8	28.6
Q63 Did you drink alcohol or use drugs before you had sexual intercourse the last time? (Yes)	19.1	12.5	14.2
**Suicide Risk**			
**Q26 During the past 12 months, did you ever seriously consider attempting suicide? (Yes)	17.7	13.4	18.0
**Q27 During the past 12 months, did you make a plan about how you would attempt suicide? (Yes)	14.5	10.4	14.8
Q28 During the past 12 months, how many times did you actually attempt suicide? (One or more times)	12.2	10.1	12.3
*Q29 If you attempted suicide during the past 12 months, did any attempt result in an injury, poisoning, or overdose that had to be treated by a doctor or nurse? (Yes)	4.2	1.8	5.1

*p < 0.05.

**p < 0.001.

### 2.3 Statistical analysis

We adjusted results for the YRBS complex survey design using the Stata (*Version 15*, College Station, TX) complex survey module. All results are presented as population-based estimates that adjust standard errors accordingly. To establish a high-risk threshold, we used 1997 data for the highest risk 20% of CPS respondents, which corresponded to students who responded affirmatively on 10 or more of the 29 items. While this cut off is empirical, the 1997 data provide a useful benchmark to identify trends in the students most likely to be experiencing serious, long-term health risks. We first present changes in the four domain mean scores across survey waves. Chi square tests were used to compare the proportion of affirmative responses on each of the 29 items across the three survey waves and to test the significance of changes in the proportion of respondents characterized as high multiple risk-exposed (≥10 affirmative responses) over the three survey waves. We compared the mean number of affirmative responses within each domain using multiple linear regression controlling for students’ grade, sex, and race/ethnicity. The regression model tests the significance of differences in survey wave year for each domain scale score, with linear estimates showing the magnitude of change associated with each survey year. We also estimated a logistic regression model of the likelihood of being in the high multiple risk category in 2007 and 2017 compared to the 1997 baseline, again controlling for students’ reported sex, race/ethnicity, and grade.

## 3 Results

As shown in Table 1, the YRBS data are based on 1,423, 1,118, and 1,883 respondents in the successive waves, reflecting a total weighted CPS high school population of 97,458 for 1997, 88,033 for 2007, and 73,313 for 2017. There was a marked increase in junior and Grade 12 respondents in 2017. The proportion of non-Hispanic Black respondents decreased from 47.8% in 1997 to 33.8% in 2017, reflecting a small gain in Non-Hispanic whites and a larger gain (13.3%) in Hispanic student respondents. The YRBS overall response rate was about 70% across the three survey waves (Fitzpatrick et al., 2019, Kann et al.,1997, Eaton et al., 2007).

Table 2 presents the 29 individual item responses across the three YRBS survey waves. There were statistically significant decreases in responses to five out of six violence/victimization questions over the period (not going to school due to feeling unsafe decreased over time but was not significant). Five of 14 substance use questions showed a significant decrease over time. These five questions related to cigarette smoking and alcohol. However, there were small, non-significant increases in cocaine use, steroids, and IV drug use (from 2.4% in 1997 to 4.1% in 2017). The proportion of students reporting starting marijuana use before 13 years decreased, although not significantly, while the proportion of ever and current marijuana users was stable over time. Significant decreases occurred in four out of five sexual health risk questions with the only exception being substance use before sexual activity. Considering suicide, making a plan, and reporting an attempted suicide needing treatment by a medical professional decreased from 1997 to 2007. However, these responses increased to above the 1997 level by 2017. Attempting suicide was not statistically different across waves but showed a similar trend. Pairwise comparison of significant chi square relationships were significant for all questions comparing 2007 to 2017, and all questions comparing 1997 to 2017 except for those accessing change in suicide risk. Only 7 out of 17 questions comparing 1997 to 2007 were significant, as questions related to sexual risk, current or ever drank alcohol, and physical fighting, injured with a weapon, and carried a weapon were not significant between only these two time periods.

### 3.1 Change in risk domain scores

As seen in Table 3, linear regression results controlled for students’ grade, sex, and race/ethnicity showed significant improvement from 1997 in three domains, but not for suicide risk. There was a –0.47 point decrease in 2017 from a 1997 mean of 1.17 affirmative responses on violence and victimization items and −0.50 point decrease in 2017 from a 1997 mean of 1.18 affirmative responses on sexual health risk items. When comparing 2017 to 1997, there was an approximate 40% reduction in affirmative responses to violence and victimization items, a 40% decline in affirmative sexual health items, and an approximate 30% decline in affirmative responses to substance use items (*p* < 0.001). While suicide risk items showed an initial approximate 25% decline in affirmative responses between 1997 and 2007, affirmative responses increased back to the mean 1997 level by 2017.

**Table 3:** Linear Regression Results Comparing 1997 Chicago Public School High School Student Responses from the Youth Risk Behavior Study to 2007 and 2017a.

	**Annual Means**	**B**	**SE**	**p**
**Violence and Victimization (6 Items)**				
1997	1.17	Reference		
2007	1.03	−0.14	0.08	0.10
2017	0.66	−0.47	0.08	<0.001
**Substance Use (14 Items)**				
1997	3.42	Reference		
2007	3.14	−0.31	0.12	0.02
2017	2.38	−1.16	0.14	<0.001
**Sexual Health (5 Items)**				
1997	1.18	Reference		
2007	1.08	−0.13	0.06	0.04
2017	0.67	−0.50	0.08	<0.001
**Suicide Risk (4 Items)**				
1997	0.45	Reference		
2007	0.33	−0.11	0.03	<0.05
2017	0.46	0.006	0.03	0.86

a Controlled for students’ grade, sex, and race/ethnicity.

### 3.2 Change in proportion of students reporting multiple risk exposure

**Fig. 1:**
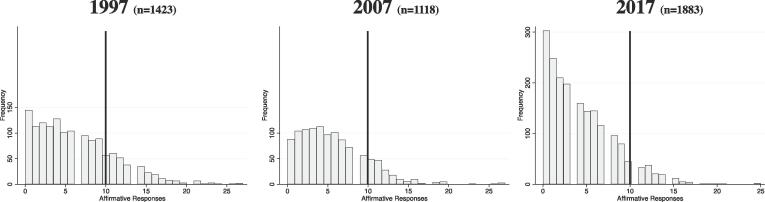
Distribution of the Number of Affirmative Responses to 29 Youth Risk Behavior Survey Questions by Chicago Public High School Students in 1997, 2007, and 2017: Proportion ≥ 10 are Highest.

The distribution of the 29-item scale scores for each year are presented in Fig. 1. The histograms show a large shift towards students reporting zero risk on the 29 items and large reductions in the number of the highest risk students. As seen in Table 4, the proportion of students reporting high multiple risk decreased from the 23.3% 1997 benchmark to 17.4% of respondents in 2007 to only 9.4% of CPS respondents in 2017 (60% reduction). The logistic regression results show that as compared to 1997 students, Chicago high school students were 30% less likely to be in the high multiple risk category in 2007 (OR 0.68; 0.50–0.91) and about 70% less likely in 2017 (OR 0.33; CI 0.22–0.49). Male students (OR 1.35; CI 1.15–1.59) were significantly more likely to be in the high multiple risk category compared to females. There were no significant differences by grade or between non-Hispanic white and non-Hispanic Black or Hispanic students. However, other/unknown race/ethnicity students (who were between five and nine percent of CPS respondents) were significantly more likely to have 10 or more affirmative answers than non-Hispanic white students (OR 0.55; CI 0.37–0.82).

**Table 4:** Logistic Regression Results for Likelihood of High Multiple Risk Respondents (Defined as ≥ 10/29 Affirmative Answers) for Chicago Public High School Students in the 1997, 2007, and 2017 Youth Risk Behavior Survey.

	Percent High Risk in 1997	Percent High Risk in 2007	Percent High Risk in 2017	**High Risk OR (95% CI)**
**2017**			9.4	0.33 (0.22–0.49)
**2007**		17.4		0.68 (0.50–0.91)
**1997**	23.3			Reference
**Male**	12.6	9.4	4.5	1.35 (1.15–1.59)
**Female**	10.6	8.0	4.6	Reference
**Non-Hispanic Black**	11.8	6.7	2.7	0.72 (0.52–1.00)
**Hispanic**	7.7	7.4	4.5	0.82 (0.55–1.22)
**Other/Unknown Race/Ethnicity**	1.9	0.8	0.8	0.55 (0.37–0.82)
**Non-Hispanic White**	1.9	2.5	1.5	Reference
**Grade 10**	6.5	4.4	2.6	0.91 (0.64–1.28)
**Grade 11**	4.4	3.3	2.0	0.79 (0.58–1.08)
**Grade 12**	4.0	3.4	2.5	1.00 (0.71–1.39)
**Grade 9**	8.3	6.3	2.1	Reference

## 4 Discussion

Study findings document significant improvement in Chicago public high school students’ long-term health risk exposure over the 20-year study period. There were significant reductions in affirmative responses to violence and victimization, sexual health, and substance use, with the biggest reductions in use of legal substances. The big exception to this trend was suicide risk. Despite an initial improvement in affirmative responses from 1997 to 2007, the YRBS suicide risk responses show an equal increase from 2007 to 2017. Using a 1997 benchmark of 10 affirmative responses across the 29 items, this study demonstrated a reduction in the proportion of students reporting very high multiple risk exposure.

### 4.1 Substance use

Since the 1990s, there has been a national decrease in teenage substance use, including cigarettes, alcohol, and select illegal and injected drugs, shown both by the YRBS and other survey data (Kann et al., 2017, Miech et al., 2019). We found similar improvements in cigarette smoking, consistent with other Chicago data (Fitzpatrick et al., 2019, Illinois Youth Survey, 2010-2018). The decrease in cigarette smoking is considered a public health success which can be attributed to multi-layered national, state, and local program activities including: legislation around raising the minimum age to buy tobacco; increasing taxes; prohibiting use in indoor public places and worksites; adverse publicity aimed at the tobacco industry; a reduction in cigarette advertising; and an increase in antismoking campaigns for youth and adults (U.S. Department of Health and Human Services. The Health Consequences of Smoking—50 Years of Progress: A Report of the Surgeon General. Atlanta: U.S. Department of Health and Human Services, Centers for Disease Control and Prevention, National Center for Chronic Disease Prevention and Health Promotion, Office on Smoking and Health, 2014). Alcohol use also significantly decreased during the study period, which is consistent with Chicago, national, and even global data (Kann et al., 2017, Miech et al., 2019, Illinois Youth Survey, 2010-2018, Pape et al., 2018). Perceived risk and disapproval seem to have played a significant role in the decrease of these substances (Miech et al., 2019, Illinois Youth Survey, 2010-2018, Pape et al., 2018).

However, the decline in smoking has been accompanied by a major increase in use of electronic vapor products, and marijuana use continues to remain prevalent (Kann et al., 2017). We were unable to measure electronic vapor use because questions regarding the use of these products were just added in 2015 (Kann et al., 2017). There was no significant change in marijuana use over time in our study, consistent with Illinois state data from 2008 to 2018 (Illinois Youth Survey, 2010-2018). In the national YRBS, the prevalence of having ever used marijuana and current marijuana use increased from 1991 to 1997 and then decreased from 1997 to 2017 (Kann et al., 2017). Daily and lifetime marijuana use in 12th graders is near the high levels recorded during the 1990s but lower than the peak in the 1970s (Miech et al., 2019). There is a changing national climate of easier recreational access and a corresponding decrease in perceived risk and perceived adult disapproval (Kann et al., 2017, Illinois Youth Survey, 2010-2018).

The trend in opioid use for teens in Chicago remains unclear. There was a small, non-significant increase in students reporting intravenous drug use in our study of Chicago YRBS data. However, the YRBS does not include questions about heroin without a needle and did not ask about misuse of prescription medications until 2017 (Kann et al., 2017). Nationally the YRBS shows a decrease in intravenous drug use, and the Monitoring the Future National Survey (MTF) sponsored by the National Institute on Drug Abuse at the National Institutes of Health describes a national decrease in opioid use, including heroin (both with and without a needle) and misuse of prescription medication, among teenagers since 2010 (Miech et al., 2019, Substance Abuse and Mental Health Services Administration. Key substance use and mental health indicators in the United States: Results from the, 2016). Also, there has been a reported decrease in the percentage of students who have used other illegal drugs, including cocaine, methamphetamines, hallucinogens, inhalants, and ecstasy (Kann et al., 2017, Miech et al., 2019). Contrary to this, there has been a disturbing increase in drug overdose deaths including opioids in teenagers since 1999 (Curtin et al., 2017). According to the CDC, the death rate due to drug overdose among teenagers aged 15–19 more than doubled from 1999 (1.6 per 100,000) to 2007 (4.2), declined by 26% from 2007 to 2014 (3.1), and then increased in 2015 (3.7), with most deaths due to opioids, specifically heroin (Curtin et al., 2017). This may represent a small very high-risk population, or perhaps the rates of youth opioid use may be higher than reported.

### 4.2 Sexual health and teenage pregnancy

There has been a remarkable national decrease in teen pregnancies, abortions, and births. Teen childbearing reached a record low of 8.8 births per 1,000 females aged 15–19 in 2017, down 55% since 2007 (Kann et al., 2017, Hamilton et al., 2017, Kost et al., 2013, Lindberg et al., 2016, Santelli et al., 2007). The decrease in sexual health risk among CPS students correlates with the remarkable decrease in teenage pregnancies and teen births in Chicago and nationally (Fitzpatrick et al., 2019, Hamilton et al., 2017, Kost et al., 2013, Lindberg et al., 2016, Santelli et al., 2007). This change has been attributed to both decreased sexual activity and increased birth control use, including long-acting reversible contraception (LARC) (Lindberg et al., 2016, Santelli et al., 2007). We found a significant 1997 to 2017 decrease in four of five questions related to sexual activity in our study, mirroring the decrease in the prevalence of all six YRBS sexual risk behaviors nationally (Kann et al., 2017). Despite these overall trends, chlamydia and gonorrhea rates are persistently rising nationally and in Chicago, and young people comprise the majority of new cases (Chicago Department of Public Health. HIV/STI Surveillance Report, 2017, Centers for Disease Control and Prevention. Sexually Transmitted Disease Surveillance, 2017). This may be related to national YRBS findings of decreased condom use from 2005 to 2017, despite an overall increase in use from 1991 to 2017 (Kann et al., 2017).

### 4.3 Violence & victimization

Chicago YRBS data are also consistent with the national long-term decline in youth violence since the 1990s (The Drug Enforcement Administration (DEA), 2019, Perlus et al., 2014). These trends reflect major reductions in violent crime for both youth and adults in American cities, including Chicago, since 1990 (Sharkey, 2018, Centers for Disease Control and Prevention, 2013). This poorly understood and under-reported decrease in urban crime has many theories including the effect of increased community activism, public and private policing, and the decrease in lead exposure, a neurotoxin linked to impulsivity, delinquent behaviors, and violence, which was removed from gasoline in the 1970s (Sharkey, 2018).

Despite this overall decrease in crime and youth violence, violence is still a major problem in Chicago, primarily for young African American men. In 2016, during a spike in total homicides, Chicago’s overall adolescent firearm homicide rate was about three times the national rate, with Chicago’s African American male adolescent firearm homicide rate nearly 50 times the national rate (Violent, 2019). The root of this violence includes current and historical institutional and structural racism, resulting in multi-generational neighborhood disinvestment, oppression, and disadvantage. Many CPS students come from neighborhoods that have suffered from the toxic stress of poverty, racism and segregation, police brutality with subsequent loss of trust, and a history of violent crime (Acs et al., 2017, Pendall, 2018). These experiences of toxic stress are embodied and can drive lasting physiological and psychological vulnerability manifesting in a myriad of health risk behaviors and poor health outcomes, including further violence and victimization (Whitfield et al., 2003, Theall et al., 2017).

### 4.4 Suicide risk

While responses to suicide risk items showed improvement among CPS respondents from 1997 to 2017, these gains were entirely reversed by 2017, reflecting nationwide trends (Fitzpatrick et al., 2019, Stone et al., 2018). Despite improvements in many other indicators of teenage well-being, teenage depression, anxiety, and suicide rates are rising (Fitzpatrick et al., 2019, Substance Abuse and Mental Health Services Administration. Key substance use and mental health indicators in the United States: Results from the, 2016, Stone et al., 2018). Teenage depression has not only been increasing but also largely goes untreated. Of the 3.1 million teenagers aged 12 to 17 who met the criteria for a major depressive episode in 2016, only about 1.2 million, or 40 percent, received treatment for depression (Substance Abuse and Mental Health Services Administration. Key substance use and mental health indicators in the United States: Results from the, 2016). After stable trends from 2000 to 2007, suicide rates for persons aged 10–24 increased from 2007 (6.8 per 100,000 persons) to 2017 (10.6 per 100,000) (Hale and Viner, 2016) and remain a leading cause of death for youth (Stone et al., 2018).

The etiology of worsening of suicide risk and mental health is likely multifactorial, with biological, psychological, interpersonal, environmental, and societal influences that interact with one another (Stone et al., 2018). These factors likely include an overall weakening in social connectedness, insufficient sleep, and an increased pressure to achieve. The current lack of social connectedness may be related to the omnipresence of teen social media and cellphone use, which ironically increases the number of social connections but may reduce their quality (Dulaney et al., 2018). Access to mental health care remains a problem, especially in many lower income Chicago neighborhoods (Dircksen, et al., 2016).

### 4.5 The decline in very high risk students

CPS students reporting high multiple risk exposure, as shown by the 10 affirmative response threshold, decreased by almost 60% from 1997 to 2017. Males remain over-represented among the highest risk. This decrease in the overall rate of high multiple risks reinforces perceptions of an increase in “conservative behavior” among teenagers nationally. This reflects changes in teen socialization and culture, as well as other possible factors such as stricter parenting (Borodovsky and Krueger, 2019). In addition, the population studied in 2017 may be very different from the population in the 1990s, as discussed below.

### 4.6 Changes in the Chicago high school population

Underlying changes in YRBS responses may mirror a change in the CPS high school population being surveyed. CPS total grade and high school enrollment has fallen from a peak of 434,00 in 2003 to 364,000 in 2018. At the same time, the increase in grade 11 and grade 12 respondents in 2017 reflects an approximate 50% increase (from about 50–75%) in the high school completion rate during this period (Hamilton et al., 2017). There was a notable decrease of about 14% in the number of non-Hispanic Black students between 2007 and 2017 with an offsetting large increase in Hispanic students, reflecting demographic changes in Chicago during the decade (Kost et al., 2013, Lindberg et al., 2016), which may have contributed to the observed reduction in risks. While the decrease in CPS students’ risk correlates with changes in the demographic composition of CPS, improvements in risk behavior and exposure occurred across every demographic subgroup. The 2017 results align with recent academic achievement gains among CPS students, including a higher graduation rate, improved test scores, and an increase in scholarship dollars received since 2011 (Schools and CPS, 2020, Reardon and Hinze-Pifer, 2017). These changes may echo and be synergistic with improvements in self-reported health risk exposure.

### 4.7 Limitations

The YRBS only surveys students, which creates a possible selection bias for lower risk teens and consideration of the possibility that attending school serves as a protective factor. While multiple risk exposure improved in this study, many adolescents not included in this study remain at very high risk for poor outcomes, including those involved with the juvenile justice system and those who are non-students and have dropped out of school.

We chose to highlight change over 20 years using only the three 10-year waves, rather than using time series analyses based on the biannual surveys. This was done for simplicity of presentation, and while biannual data might provide historical texture, it is unlikely our overall findings would differ much. The weighted YRBS estimates are also somewhat lower than published CPS enrollment demographics (Schools and CPS, 2020) but otherwise were consistent with CPS demographic data. The 29 items we chose to analyze do not represent the complete range of behavioral, environmental, and health questions on the full YRBS but do have the advantage of identical wording over the three decades, good test–retest reliability, and inclusion of public health domains rooted in childhood experience with huge implications for adult health. While the cutoff used for highest risk students was arbitrary, use of a different cutoff would not have changed the finding that there are many fewer such individuals in 2017 than in 1997.

## 5 Conclusion

In conclusion, Chicago is aligned with the nation in documenting major improvements in teen health risk exposure over the last 20 years for substance use, sexual health, and violence and victimization. These results, while providing some encouragement to advocates of adolescent health, also call attention to worsening suicide risk and the impact of vulnerability factors, such as childhood adversity and unmet social needs. Increased CPS funding is needed to assist educators, clinicians, and families in meeting basic social needs, supporting parents, ensuring multiple caring adults for all children, promoting restorative justice changes to disciplinary procedures, and improving access to school-based health services, including mental health care and counseling in schools. Equitable investment in schools increases the capacity to support vulnerable students both inside and out of the classroom.

Support: This research did not receive any specific grant from funding agencies in the public, commercial, or not-for-profit sectors.

## CRediT authorship contribution statement

**Jacqueline Korpics:** Conceptualization, Methodology, Formal analysis, Writing - original draft, Writing - review & editing. **Audrey Stillerman:** Writing - review & editing. **Keiki Hinami:** Writing - review & editing. **Sadhana Dharmapuri:** Writing - review & editing. **Joseph Feinglass:** Supervision, Conceptualization, Methodology, Formal analysis, Writing - original draft, Writing - review & editing.

## Declaration of Competing Interest

The authors declare that they have no known competing financial interests or personal relationships that could have appeared to influence the work reported in this paper.
